# Neither ant dominance nor abundance explain ant-plant network structure in Mexican temperate forests

**DOI:** 10.7717/peerj.10435

**Published:** 2020-12-07

**Authors:** Brenda Juárez-Juárez, Mariana Cuautle, Citlalli Castillo-Guevara, Karla López-Vázquez, María Gómez-Ortigoza, María Gómez-Lazaga, Cecilia Díaz-Castelazo, Carlos Lara, Gibrán R. Pérez-Toledo, Miguel Reyes

**Affiliations:** 1 Maestría en Biotecnología y Manejo de Recursos Naturales, Centro de Investigación en Ciencias Biológicas, Universidad Autónoma de Tlaxcala, San Felipe Ixtacuixtla, Tlaxcala, Mexico; 2 Departamento de Ciencias Químico Biológicas, Universidad de las Américas Puebla, San Andrés Cholula, Puebla, Mexico; 3 Centro de Investigación en Ciencias Biológicas, Universidad Autónoma de Tlaxcala, San Felipe Ixtacuixtla, Tlaxcala, Mexico; 4 Red de Interacciones Multitróficas, Instituto de Ecología A.C., Xalapa, Veracruz, Mexico; 5 Red de Ecología Funcional, Instituto de Ecología, A.C., Xalapa, Veracruz, Mexico; 6 Departamento de Actuaría, Física y Matemáticas, Universidad de las Américas Puebla, San Andrés Cholula, Puebla, Mexico

**Keywords:** Nestedness, *Camponotus rubrithorax*, *Formica* spp., *Prenolepis imparis*, Oak forest, Grassland, Ant hierarchy dominance

## Abstract

**Background:**

Ant-plant mutualistic networks tend to have a nested structure that contributes to their stability, but the ecological factors that give rise to this structure are not fully understood. Here, we evaluate whether ant abundance and dominance hierarchy determine the structure of the ant-plant networks in two types of vegetation: oak and grassland, in two temperate environments of Mexico: Flor del Bosque State Park (FBSP) and La Malinche National Park (MNP). We predicted that dominant and abundant ant species make up the core, and submissives, the periphery of the network. We also expected a higher specialization level in the ant trophic level than in plant trophic level due to competition among the ant species for the plant-derived resources.

**Methods:**

The ant-plant interaction network was obtained from the frequency of ant-plant interactions. We calculated a dominance hierarchy index for the ants using sampling with baits and evaluated their abundance using pitfall traps.

**Results:**

In MNP, the *Formica* spp. species complex formed the core of the network (in both the oak forest and the grassland), while in FBSP, the core species were *Prenolepis imparis* (oak forest) and *Camponotus rubrithorax* (grassland). Although these core species were dominant in their respective sites, they were not necessarily the most dominant ant species. Three of the four networks (oak forest and grassland in FBSP, and oak forest in MNP) were nested and had a higher number of plant species than ant species. Although greater specialization was observed in the ant trophic level in the two sites and vegetations, possibly due to competition with the more dominant ant species, this was not statistically significant. In three of these networks (grassland and oak forest of MNP and oak forest of FBSP), we found no correlation between the dominance hierarchy and abundance of the ant species and their position within the network. However, a positive correlation was found between the nestedness contribution value and ant dominance hierarchy in the grassland of the site FBSP, which could be due to the richer ant-plant network and higher dominance index of this community.

**Conclusions:**

Our evidence suggests that ant abundance and dominance hierarchy have little influence on network structure in temperate ecosystems, probably due to the species-poor ant-plant network and a dominance hierarchy formed only by the presence of dominant and submissive species with no intermediate dominant species between them (absence of gradient in hierarchy) in these ecosystems.

## Introduction

The study of mutualistic interactions between plants and animals is essential to the understanding of ecological systems. Understanding these systems is necessary to develop effective management and conservation methods for their biodiversity (Rico-Gray & Oliveira, 2007). Ant-plant mutualisms are characterized by the association between a plant that provides a resource (i.e., extrafloral nectar, floral nectar, seeds) or shelter (domatia) and ants that offer services to the plant. These services include (1) herbivory reduction (Bronstein, Alarcón & Geber, 2006; Rico-Gray & Oliveira, 2007; Mayer et al., 2014), (2) seed dispersal (Cuautle, Rico-Gray & Díaz-Castelazo, 2004; Salazar-Rojas et al., 2012) and (3) pollination (Puterbaugh, 1998; Del-Claro et al., 2019). Of the three, herbivory reduction is undoubtedly the most important and most commonly occurring service that ants provide to plants, while the second, seed dispersal, is significantly less common. The third, pollination, is close to negligible when all known ant species and plants potentially pollinated by ants are considered. However, this service may be more prevalent in alpine and arid environments with low species richness (Puterbaugh, 1998; Del-Claro et al., 2019). Ant-plant mutualisms can also be mediated indirectly, such as when Hemiptera feed on a plant’s sap and excrete honeydew, which is then foraged by the ants (Bronstein, Alarcón & Geber, 2006; Rico-Gray & Oliveira, 2007; Mayer et al., 2014). Studies of plant-ant mutualisms are being carried out at the community level, using a common tool to study complex networks, graph theory (Bascompte et al., 2003). In these graphs, a set of nodes and lines joining the nodes represent the different species and their interactions. The study of different types of mutualistic networks (e.g., plant–pollinator, plant-frugivore, plant-disperser) has shown that these networks are highly heterogeneous (i.e., most species have few interactions, but a few species are much more connected than what would be expected by chance). This heterogeneity renders a nested and asymmetric topology where specialists interact with subsets of the species with which generalists interact (Bascompte & Jordano, 2007). This nested structure is of crucial importance because it increments community stability. It promotes higher resilience to extinction for the core of generalist species, as well as the specialist species that are linked to these generalists (Dehling, 2018).

While the study of mutualistic interaction networks in biological systems has increased our understanding of their organization (Bascompte et al., 2003), recent studies have aimed to identify the ecological factors that determine the nested structure in these networks (Memmott et al., 2007; Santamaría & Rodríguez-Gironés, 2007). In the case of associations between ants and plants with extrafloral nectaries (EFN), several factors have been identified that may contribute to the nested structure of the networks in tropical ecosystems. (1) abiotic factors: monthly precipitation and temperature increase nestedness (Rico-Gray et al., 2012). (2) body size: ant size and degree of nestedness are positively correlated (Chamberlain, Kilpatrick & Holland, 2010). (3) extrafloral nectary phenology: during the months of highest activity in the nectaries, there is greater nestedness (Lange, Dáttilo & Del-Claro, 2013). (4) species richness: species-rich systems are highly nested (Guimarães Jr et al., 2006) and (5) ant dominance hierarchy: dominant ants are found at the core of the network (Dáttilo et al., 2013). However, most of these studies were carried out in tropical ecosystems. Interestingly, one of the few studies that involved a temperate ecosystem found that the network involving EFN-bearing plants and ants was not nested. The authors attributed this finding to low species richness in these environments (Guimarães Jr et al., 2006). A central aspect in which temperate and tropical ecosystems differ is that of ant richness. Tropical ant communities can be composed of hundreds of ant species (Arnan, Gaucherei & Andersen, 2011; García-Martínez et al., 2015); whereas temperate ant communities are typically constituted by 20 species or fewer (Lynch & Johnson, 1988; Cuautle, Vergara & Badano, 2016). Therefore, it is reasonable to assume that ant species richness may be a factor shaping ant-plant interaction richness, which affects the network structure.

Competition has been largely ignored in studies of mutualistic networks involving ants and EFN-bearing plants (Chamberlain & Holland, 2009; Dáttilo et al., 2014a). However, the dominance hierarchy (i.e., ordering of ant species based on their numerical or behavioral dominance, Stuble et al., 2017) plays a significant role in the structuring of the nested pattern in networks involving tropical ants and EFN-bearing plants (Dáttilo, Díaz-Castelazo & Rico-Gray, 2014). Thus, the generalist ants of the core (dominant) are competitively superior to the peripheral ant species (submissive). This structure confers stability to the composition of the core species over vast geographic distances, during all phenological stages of nectar secretion or even after disturbances caused by hurricanes (Díaz-Castelazo et al., 2013; Lange, Dáttilo & Del-Claro, 2013; Dáttilo et al., 2013). On the other hand, it is known that core ant species interact with plants more than would be expected from their local abundance, suggesting that their abundance only partly explains the probability that these generalist ant species find their food resource (Dáttilo et al., 2014b).

The few studies carried out in Mexico that focus on the ecological factors determining the asymmetrical nested topology of ant-plant interaction networks, such as abundance and dominance hierarchy (Dáttilo, Díaz-Castelazo & Rico-Gray, 2014), took place in tropical environments. Currently, little is known about the ant-plant interactions and the ecological factors that structure the networks in temperate ecosystems (but see Guimarães Jr et al., 2006). Here, we evaluate whether the ant-plant network structure of two temperate ecosystems, in two types of vegetation, is determined more by dominance hierarchy (a ranking of the ant species based on its numerical dominance) than abundance. Our study aimed to describe (1) the structure of the networks, (2) specialization asymmetry, (3) the core and peripheral ant species, (4) the dominance hierarchy and abundance of the ants, and (5) the relation between the dominance hierarchy and abundance of the ants and their position within the ant-plant network. We hypothesized that the network structure would be nested and that if ant competition were an essential factor underlying the structure, higher specialization on the ant trophic level would be present to reduce competition for resources. We also predicted that in these temperate ecosystems, dominance and abundance establish the ant species position within the network. In other words, we expected the most dominant and abundant ants would be in the core and the submissive and less abundant ones in the periphery.

## Materials and Methods

### Study sites

The study was conducted in two temperate natural protected areas: La Malinche National Park (MNP) and Flor del Bosque State Park (FBSP). The Secretaria de Medio Ambiente y Recursos Naturales (SEMARNAT) (Secretary of Environment and Natural Resources) approved the collection permit SGPA/DGV/06901/15 for this study. The MNP consists of 46,093 ha and is located between the Mexican states of Tlaxcala and Puebla (19°20′N, 98^°^09′W). The study area’s vegetation is a mosaic of coniferous and oak forest, grassland, and second-growth vegetation resulting from fire events in the original forest or abandoned agricultural areas (López-Domínguez & Acosta-Pérez, 2005). The FBSP consists of 664.03 ha and is located in Puebla (19°01′N, 98°20′W). This area’s vegetation is characterized by oak forest (41.71% of the park’s surface), eucalyptus (1.52%), and grassland (16.25%) (Costes-Quijano et al., 2006).

In both sites, six transects were established (400 m length, spaced at least 1.5 km apart), three in oak forest (OF), and three in grassland (G). In these transects, samplings were carried out to determine the ant-plant network, dominance, and abundance. In the MNP, the transects’ altitudinal range spanned 2,700–2,900 m above sea level (m a.s.l.), while in the FBSP, they were located from 2,300–2,400 m a.s.l. The two types of vegetation present at these altitudinal ranges were considered in this work.

### Data collection

#### Ant-plant network sampling

MNP’s network data was taken from a previous study by our research group (Lara et al., 2020). This study reported the ant-plant networks obtained by monthly sampling (from June 2015 to July 2016) conducted in conserved and perturbed oak forest sites. The network data derived from the two vegetation types present in the study area (oak forest and grassland) was used for the analyses described below. FBSP’s networks were acquired in 2016 by monthly sampling (from January to December 2016) of each site’s ant-plant interactions. Both of these studies were carried out during the dry and rainy seasons and were conducted with the following protocol. To avoid bias, observers were trained by a single team member (M. Cuautle) before initiating the data collection. Walking censuses were undertaken from 08:00 h to 13:00 h on days with mild weather conditions, commencing from a different paired transect and vegetation type to avoid order effects and the confounding effect of detection of interactions in the field. The same observer conducted the day’s census, taking around 1 h in each transect. Observers recorded any type of interaction between individual ants and plants (i.e., foraging for floral, extrafloral nectar or honeydew), at a distance of no more than 10 m from the centerline of the transect. In trees, ant-plant interactions were recorded up to 1.5 m above ground level. The ants were collected with an entomological aspirator and stored in Eppendorf tubes with 70% alcohol. In addition, samples were collected from the plants with which the ants interacted. Both ant and plant specimens were identified using taxonomic keys. R. Acosta-Pérez from the Universidad Autónoma de Tlaxcala, Botanical Laboratory identified the plants, and G. R. Pérez-Toledo made most of the ant determinations up to species level (using the taxonomic keys of Baroni, 1978; Mackay & Mackay, 1989; Francoeur, 1973; MacKay, 2000; Longino, 2009; Cuezzo & Guerrero, 2011; Stockan et al., 2016). W. Mackay from the University of Texas, Laboratory for Environmental Biology Centennial Museum, determined the ant *Camponotus rubrithorax*.

### Ant sampling: pitfalls and baits

Ant abundance was calculated using the frequency at which each species was recorded in pitfall traps. This method was used to avoid the overestimation of ant species that have more efficient recruitment systems (Gotelli et al., 2011). The pitfall traps were placed in the dry season (March) and rainy season (July) of 2015 at the MNP and 2017 at the FBSP. In each site, 20 pitfall traps were placed per transect (spaced 20 m apart), for a total of 240 traps (20 traps per transect × 6 transects × 2 seasons). Ninety-six hours later, the traps were collected.

To evaluate the dominance hierarchy of MNP’s ants, we used the Dominance Index (DI) data that were calculated in a previous study at the same site from April to September of 2015 (Castillo-Guevara et al., 2019). In this study, nine sampling points separated by 10 m were established in each transect. At each sampling point, a pair of Petri dishes were placed on the ground with baits, one with honey and the other with tuna (<5 cm distance), 648 baits were placed in total (6 monthly replicas × 6 transects × 9 sampling points × 2 baits). Although honey or tuna are not exact dietary equivalents for nectar or prey, they are a very well accepted source of carbohydrates and protein by ants, and these types of baits have been widely used for ant dominance hierarchy studies (Trigos Peral et al., 2016; Parr & Gibb, 2012; Dáttilo, Díaz-Castelazo & Rico-Gray, 2014). Also, honey and tuna baits are more practical when a large number of baits are used (Fellers, 1987). The ant species and the number of individuals that arrived at the baits were recorded during the observation periods (9:00 a.m. to 5:00 p.m.). The experimental design for FBSP was similar to the one described above and was carried out from October 2016 to March 2017 and July 2017. In this location, 504 baits were placed in total (7 monthly replicas × 6 transects × 6 sampling points × 2 baits). The ants collected from the pitfalls and baits were stored in 70% ethanol and taken to the laboratory for identification. The placement of the Petri dishes on the ground may represent a bias of this study, as foliage-foraging ant communities form these networks. However, placing baits on the vegetation would have introduced another bias, as dominant ants would already be monopolizing the plants’ sugar sources.

### Statistical analysis

#### Ant-plant network metrics

For each ant-plant community, interactions were summarized as a bipartite matrix, consisting of cells filled with the pairwise interaction frequency of all ant and plant species. Separate analyses were performed for each vegetation community (oak forest and grassland) in both areas (MNP and FBSP). Given the similarities found in the oak forest and the grassland of the MNP (three of six shared ant species and eleven of thirty shared plant species), as a second approximation, data derived from both communities were pooled. This approximation increased the sample (number of ant species) used for the correlation analysis, which is worthwhile because a larger sample size can augment the test’s statistical power (Zar, 1996). The results of the analysis performed on the pooled data from the MNP communities are presented in a Table S1. The following complementary network metrics were calculated; (1) nestedness, (2) web asymmetry, (3) specialization asymmetry, (4) species strength, and (5) identification of the core and peripheral species in each ant-plant network. (1) Nestedness quantifies the degree to which interactions of specialized species are subsets of interactions of the more generalist species in the networks and was calculated with the NODF estimator of the ANINHADO software (Guimarães Jr & Guimarães, 2006; Almeida-Neto et al., 2008). The topology of a network was considered nested when the observed NODF value (NODF) was higher than predicted by the null model Er (NODF(Er)), with 1,000 randomizations for each network (Guimarães Jr et al., 2006). In the null model, (Er) interactions are randomly assigned (Bascompte et al., 2003). Web asymmetry, species strength, and specialization asymmetry were estimated with the bipartite library (Dormann, Fruend & Gruber, 2018) in the statistical software package R (R Development Core Team, 2014). (2) Web asymmetry is the difference between the number of ant and plant species in a network divided by the total number of species in that network, where positive values indicate higher-trophic-level species, while negative values denote lower-trophic-level species (Blüthgen et al., 2007). (3) Specialization asymmetry, based on the degree of specialization of each species (*d*′), does not vary significantly across networks of different dimensions. Since the mean d-value for the lower trophic level (plants) is subtracted from that of the higher level (ants), positive values indicate a higher specialization of the higher trophic level (Dormann, Fruend & Gruber, 2018). The degree of specialization can be higher or lower than expected, given the web asymmetry (Blüthgen et al., 2007). To determine if this was the case, we estimated the significance of web specialization asymmetry with a Monte Carlo procedure in which 100 random matrices were generated using the null model (Patefield algorithm), where marginal totals were identical to those of the observed network (Anjos, Dáttilo & Del-Claro, 2018). To define the role of each interacting species within the networks, we used (4) species strength (ST) that is the importance of a species for the entire network, i.e., the sum of the dependencies of all interaction partners of that species (Bascompte, Jordano & Olesen, 2006). Lastly, to identify (5) the core and peripheral species in each ant-plant network, we used the equation:

\begin{eqnarray*}& & {G}_{i}= \left( \frac{{k}_{i}-\overline{k}}{\sigma k} \right) \end{eqnarray*}

where *G*_*i*_ is species centrality; *k*_*i*_ = average number of links involving an ant species *i*; $\overline{k}$ = average number of links involving all ant species in the network, and *σ*_*k*_ is the standard deviation of the number of links of all ant species. *G*_*c*_ values >1 were core species, and species <1 were peripheral species. Core species interact with virtually all species in the network, while peripheral species have fewer interactions with other species of the same trophic level (Dáttilo, Guimarães Jr & Izzo, 2013).

### Ant abundance and dominance hierarchy indices

Ant abundance was quantified using the frequency the species was reported in the pitfall traps to avoid overestimating the species that have more efficient recruitment systems (Gotelli et al., 2011). The average numerical DI was calculated for each ant species recorded in the previous study at MNP (Castillo-Guevara et al., 2019), and analyzed de novo in FBSP. The formula used was:

\begin{eqnarray*}& & {DI}_{i}= \left( \frac{{D}_{i}}{{D}_{i}+{S}_{i}} \right) \end{eqnarray*}

where (*DI*_*i*_) is the numerical dominance index for each ant species *i*; *D*_*i*_ is the number of monopolized baits by ant species *i*, and *S*_*i*_ is the number of baits that ant species *i* used but did not monopolize. The baits were considered monopolized when >5 individuals (workers or soldiers) of the same species were using the resource without other species present. This criterion takes into account that in temperate ecosystems, ant abundance is lower than in tropical environments where the index has been used more frequently (Santini et al., 2007). The index ranges from 0 (completely submissive species) to 1 (totally dominant species) and is similar to the “monopoly index” used in other studies (Fellers, 1987; Santini et al., 2007; Parr & Gibb, 2012). The dominance hierarchy was constructed by ordering the species with the highest DI to lowest DI (Stuble et al., 2017); ants were classified as dominant with DI values greater than 0.5, and as submissive with values less than 0.5. Given the gradient in the dominance index, subdominant ants species could also be integrated into this classification. A Student’s *t*-test was performed to evaluate differences between the average DI of the core and peripheral ant species in the network’s of MNP’s oak forest and grassland and the oak forest in FBSP, as these data covered normality assumptions. While in FBSP’s grassland, a Mann Whitney test was performed, as the data was not normally distributed.

### Modified DI

We propose a new index to evaluate the dominance hierarchy of ants in our temperate ecosystems. Our proposal is based on a modification of the numerical dominance index used by Dáttilo et al. (2014b) in tropical environments, which quantifies the number of baits occupied by a single species of ant, regardless of the number of individuals of that species present at the bait. The index we use is MDI = (*Cs*∕*Cs* + *Zs*); where MDI is the modified numerical dominance, *Cs* is the number of baits occupied by the ant species without the presence of other ant species; and *Zs* is the number of baits occupied by the ant species with the presence of other ant species. The index range is from 0 (totally submissive species) to 1 (totally dominant species). The proposed index differs from that of numerical dominance because a minimum number of individuals is not required to determine that a species of ant monopolized a bait, just that it was the only ant species at the bait. The above is proposed as a way to correct a potential bias of the numerical dominance index, given the diverse recruitment strategies employed by the ants (i.e., mass, group, tandem). In the MNP and FBSP, the average MDI for each ant species was calculated by vegetation type (oak forest and grassland). A Student’s *t*-test was performed to evaluate differences between the average MDI of the network’s core and peripheral ant species, as the data covered normality assumptions.

#### Nestedness measure correlations

Nestedness measures that represent the ant’s position in the network were used to assess whether dominance hierarchy determines the nestedness scores in the ant-plant network. (1) Nestedness ranking (Nra) (Dáttilo et al., 2014b), that is, the species of ants in the network were organized according to their degree of nestedness (i.e., the number of links per species), and ordered from highest to lowest. (2) Nestedness contribution (Nc). To obtain this parameter, we used the equation Nc = (100 − IT)/100, where IT is the idiosyncratic temperature, species with high IT have an atypical pattern of interaction compared with other species of their same trophic level (Vilà et al., 2009). Because generalist species interact with many other species, their interaction pattern is similar to other species at the same trophic level, resulting in lows ITs and highs Ncs; the contrary happens with the specialist species. ITs were calculated with ANINHADO software (Guimarães Jr & Guimarães, 2006; Almeida-Neto et al., 2008). (3) Nested rank (Nr) orders species according to their generality, which is measured as their position in the nestedness matrix. A generalist will interact with more species and thus have a rank closer to 0, while specialists (and rare species) will have higher ranks (closer to 1) (Alarcón, Waser & Ollerton, 2008). Nested ranks were calculated using the bipartite library (Dormann, 2011; Dormann, Gruber & Fruend, 2008; Dormann, Fruend & Gruber, 2018) in the statistical software package R (R Development Core Team, 2014). (4) Expected frequency was calculated only for FBSP. The sample numbers from the MNP were too low to make this approximation worthwhile, as the ant’s expected frequencies would not differ from their degree of nestedness. A plot was constructed of the frequencies of the grades (i.e., the number of links) for both types of vegetation in FBSP’s network. Ideally, the frequencies plot will have a smooth, continuous estimate of frequencies at each grade. To obtain that continuous estimate, we used a nonparametric approximation: kernel density estimation. Based on a set of observations, this technique estimates the density function from which the observations may have been derived. In this case, most of the sets of frequencies taken from the observations of FBSP’s oak forest and grassland are close to zero making it difficult to obtain an accurate density estimate with this technique. To overcome this issue, we used the transformation kernel density estimator. It spreads the observations out, finds their density estimate, and then reverts them to the original scale, resulting in a distribution that fits the observed density. From here, obtaining a smooth pattern for the frequencies is straightforward, since both the frequency and density functions are the same except for a constant of proportionality. A log transformation was used to implement this methodology. This procedure is well explained and justified in Wand & Jones (1995). Subsequently, these four nestedness measures were correlated with the abundance, DI, and MDI using a Spearman correlation.

All data collection and analysis of the FBSP sampling was conducted for the present study. Whereas for the MNP, the ant-plant matrix data was compiled from Lara et al. (2020), and the dominance hierarchy ranking was taken from Castillo-Guevara et al. (2019). However, the ant abundance calculation and data analysis of MNP were carried out for this study.

## Results

### Structure of the ant-plant network

#### Ant-plant network MNP

In MNP’s oak forest, 18 interactions were observed, comprising 13 plant species and five ant species. The plant species included eight families, of which the Fagaceae family was the most represented (*N* = 5 species). For the ants, the Formicinae and Myrmicinae subfamilies were recorded, the latter being the most represented (*N* = 3 species) (Lara et al., 2020).

Given the difficulty of identifying some of the ant species in the field, *Formica* spp. Linneaus refers to the three morphospecies of the genus that are ecologically and functionally similar (Castillo-Guevara et al., 2019). Two of these species were later determined to be *F. propatula* Francoeur and *F. pacifica* Francoeur. Both ant species were found in the oak forest and grassland of the MNP. The ant-plant network of MNP’s oak forest is significantly nested (Lara et al., 2020). Five plant species (39% of the total registered species) and one ant species (*Formica* spp., 20% of the total recorded species) were identified as forming the network’s core.

In grassland, 33 interactions were observed, comprising 28 plant species and four ant species (Lara et al., 2020). The plant species included 17 families, of which Asteraceae was the most represented (*N* = 8 species) (Lara et al., 2020). For the ants, the Formicinae and Myrmicinae subfamilies were recorded, the latter being the most represented (*N* = 3 species) (Lara et al., 2020). The ant-plant network was not significantly nested (Lara et al., 2020). Three plant species (11% of the total registered species) and one ant species (*Formica* spp. 25% of the total recorded ants) were identified as forming the core of the network.

#### Ant-plant network FBSP

**Figure 1:**
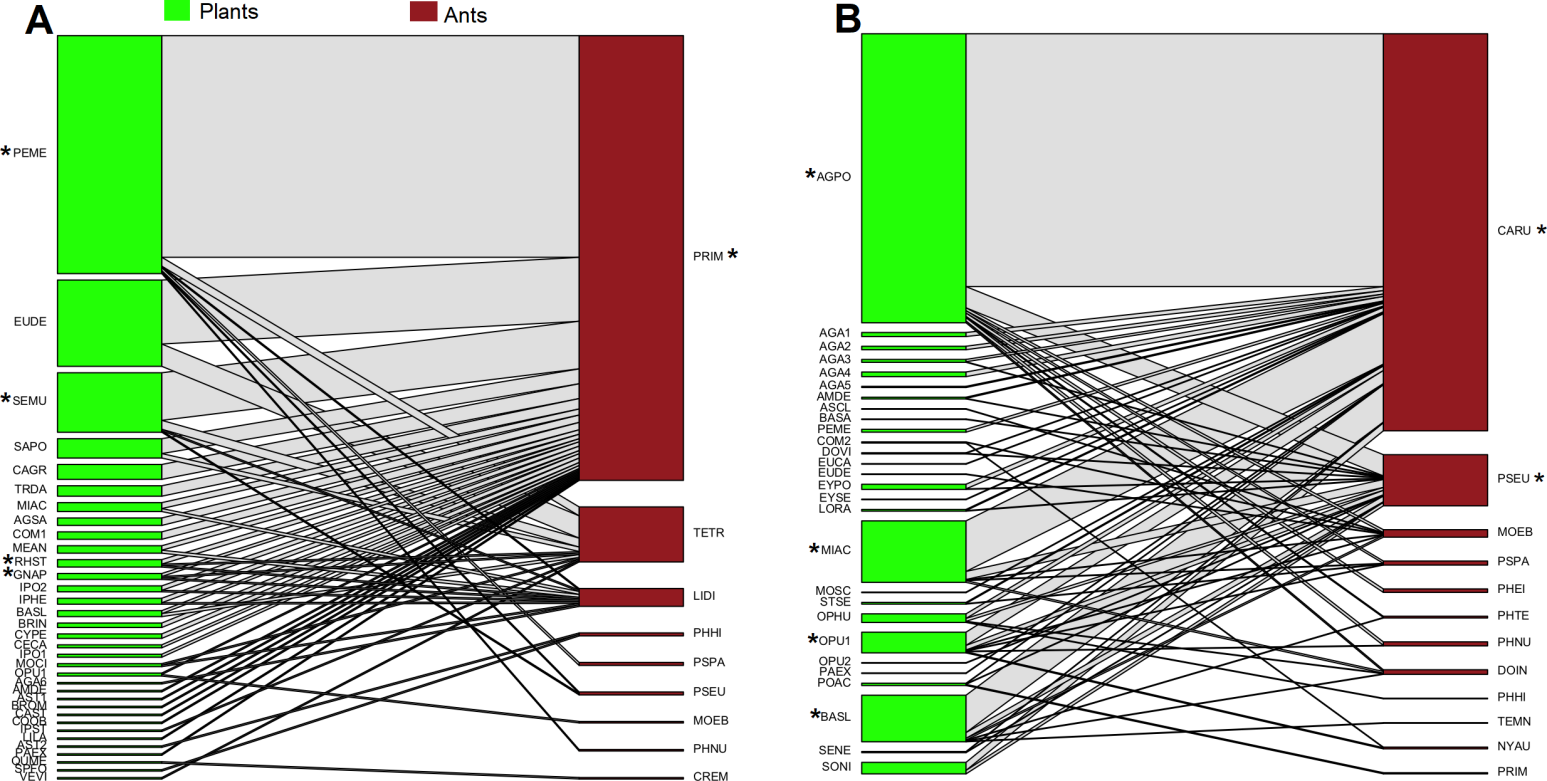
Ant-plant interaction networks in the oak forest (A) and the grassland (B). Each box represents a species of plant or ant (plants green, ants brown), and the lines represent the frequency of the ant-plant interactions. Species that were present in the core of the network are represented by an asterisk (*).

In oak forest, 55 interactions were observed, comprising 34 plant species and nine ant species (Fig. 1A, Data S2). The plant species comprised 19 families of which Asteraceae was the most represented (*N* = 8 species). For the ants, four subfamilies were recorded, and the most represented was Myrmicinae (*N* = 5 species) (see Table S2). A significantly nested pattern was observed in the ant-plant network (NODF = 43.13, NODF(Er) = 19.56, *P* < 0.01). Four plant species (12% of total recorded species) and one ant species (*Prenolepis imparis* Say, 11% of total recorded species) shape the core of the network (Fig. 1A).

In grassland, 63 interactions were observed, comprising 28 plant species and 12 ant species (Fig. 1B, Data S2). The plant species comprised 14 families of which Asteraceae and Agavaceae were the most represented (*N* = 6 species each). For the ants, four subfamilies were recorded, and the most represented was Myrmicinae (*N* = 6 species) (see Table S2). A significantly nested pattern was observed in the ant-plant network (NODF = 57.91, NODF(Er) = 20.75, *P* < 0.01). Four plant species (14% of total recorded species) and two ant species (*Camponotus rubrithorax and Pseudomyrmex* sp. 17% of total recorded species) shape the core of the network (Fig. 1B).

### Network parameters (trophic and species level)

Overall, similar patterns were found in the evaluated network parameters at both study sites (MNP and FBSP), even though the ant and plant species were different in each network. The asymmetry of the networks show a greater number of plants available for the ants (web asymmetry: MNP, Oak forest = −0.44, Grassland = −0.77 see Lara et al., 2020; FBSP, Oak forest = −0.58, Grassland = −0.40), and the ants were more specialized than the plants, although this specialization was not significantly different than what would be expected randomly (specialization asymmetry: MNP, Oak forest = 0.47, Grassland = 0.33; FBSP, Oak forest = 0.13, Grassland = 0.63, *P* > 0.07). In both types of vegetation in the MNP, *Formica* spp. had the highest species strength (Lara et al., 2020). In the oak forest of the FBSP network, *P. imparis* (species strength = 23.10) had the highest species strength and in the grassland, *C. rubrithorax* (species strength = 18.31). The most relevant plant in the MNP network, with the highest species strength value, was *Barkleyanthus salicifolius* (Kunth) H. Rob. & Brettell, in both vegetation types (species strength: Oak forest = 16.76, Grassland = 2.65, Lara et al., 2020). In the FBSP’s networks, the most important plant species with the highest species strength were *Agave potatorum* Zuccarini (species strength = 4.91) in the grassland and *Perymenium mendezii* D. C. (species strength = 3.24) in the oak forest.

### Abundance and dominance hierarchy

#### Ant abundance MNP

Only 200 of the 240 pitfall traps placed in the MNP were effective (i.e., 40 were damaged by animals or people). In total, 18 morphospecies (13 species and five genera) were registered, of which *Formica* spp. had the highest abundance in the oak forest (44 occurrences, i.e., number of times the ant was recorded) and the grassland (97 occurrences) (Table 1). One species was found exclusively in the oak forest (*Lasius latipes* Walsh), while three morphospecies were exclusive to the grassland (*Dorymyrmex* sp. Mayr, *Brachymyrmex* sp. Mayr, and *Acanthomyops* sp. Mayr). Fourteen of the registered morphospecies were shared by both types of vegetation (*Formica* spp., *Temnothorax puctithorax* Mackay, W.P., *Temnothorax neomexicanus* Wheeler, W.M., *Temnothorax texanus* Wheeler, W.M., *Lasius niger* Linnaeus, *Pheidole soritis* Wheeler, W. M., *Pheidole chalca* Wheeler, W.M., *Pheidole* sp. 1 Westwood, *Myrmecocystus melanoticus* Wheeler, W.M., *Crematogaster lineolate* Say, *Monomorium minimum* Buckley, *Myrmica Mexicana* Wheeler, W. A., *Camponotus picipes pilosulus* Emery and *Solenopsis picea* Emery)

**Table 1:** Nestedness measures, abundance and dominance indices of the ant species at the Malinche National Park (MNP) and Flor del Bosque State Park (FBSP), in both types of vegetation: oak forest (OF) and the grassland (G).

Nra	Nc	Nr	Ef	A	*DI*	*MDI*	Baits occupied (only species present)
**Ant species MNP OF**								
*Formica* spp.^*^	1	1	0	–	44	0.04 ± 0.08, 6	0.78 ± 0.08,6	62 (48)
*Temnothorax punctithorax*	2	0.83	0.75	–	13	0.05 ± 0.08, 3	0.16 ± 0.17,3	15 (3)
*Myrmica mexicana*	3	0.97	0.5	–	39	0 ± 0, 2	0 ± 0,2	2 (0)
**Ant species MNP G**								
*Formica* spp.^*^	1	0.99	0	–	97	0.07 ± 0.05, 6	0.61 ± 0.14, 6	131 (80)
*Temnothorax punctithorax*	2	0.95	0.33	–	57	0 ± 0, 6	0.19 ± 0.16, 6	56 (12)
*Monomorium minimum*	3	1	1	–	29	0.31 ± 0.35, 6	0.43 ± 0.34, 6	35 (17)
**Ant species FBSP OF**								
Prenolepis imparis^*^	1	0.98	0	925	71	0.28 ± 0.21,7	0.60 ± 0.21,7	29 (19)
*Linepithema dispertitum*	2	0.91	0.25	4929	22	0.27 ± 0.21,4	0.65 ± 0.41,4	14 (9)
*Pheidole hirtula*	3	0.88	0.37	18263	60	0 ± 0,4	0.25 ± 0.50,4	6 (1)
*Monomorium ebenium*	4	0.98	0.75	39816	10	0.39 ± 0.41,6	0.74 ± 0.31,6	27 (20)
**Ant species FBSP G**								
Camponotus rubrithorax^*^	1	0.97	0	13.81	41	0.05 ± 0.13,7	0.83 ± 0.16,7	32 (27)
*Monomorium ebenium*	2	0.94	0.18	45.12	13	0.12 ± 0.16,5	0.52 ± 0.38,5	14 (8)
*Dorymyrmex insanus*	3	0.99	0.27	140.23	6	0.33 ± 0.58,3	0.58 ± 0.52,3	7 (5)
*Pheidole tepicana*	4	0.99	0.54	285.66	1	0.15 ± 0.30,4	0.75 ± 0.28,4	14 (10)
*Pheidole nubicola*	5	0.99	0.45	285.66	15	0 ± 0,1	1 ± 0,1	1 (1)
*Nylanderia austroccidua*	6	0.95	0.63	285.66	0	0 ± 0,1	0 ± 0,1	1 (0)
*Pheidole hirtula*	7	0.96	0.91	397.09	9	0 ± 0,3	0.56 ± 0.50,3	6 (4)

**Notes.**

Nestedness Ranking (Nra), Nestedness Contribution (Nc), Nested Rank (Nr), Expected frequency (Ef), Abundance (A), Dominance Index (*DI*) (average ± standard error, *n*), Modified Dominance Index (*MDI*) (average ± standard error, *n*) and no. of baits occupied by the species and (no. of baits where it was the only species present).

* Core species of the ant-plant interaction network. The *DI* and *MDI* range from 0–1; values < 0.5 = submissive species, > 0.5 = dominant species.

Expected frequencies were not calculated for MNP due to the low number of sample.

#### Ant abundance FBSP

In the FBSP, only 226 of the 240 pitfall traps that were placed were effective. In total, 25 morphospecies (10 species and 15 genera) were registered. In the oak forest, *Prenolepis imparis* had the highest abundance (71 occurrences), and in the grassland was *Camponotus rubrithorax* (41 occurrences) (Table 1). Twenty-five morphospecies were recorded, of which seven were exclusive to the oak forest (*Prenolepis imparis*, *Neivamyrmex* sp. 1 Borgmeier, *Myrmica mexicana*, *Pheidole* sp. 5, *Temnothorax andrei* Emery, *Stenamma manni* Wheeler, W. M. and *Camponotus* sp. 3 Mayr), and seven exclusive to the grassland (*Camponotus rubrithorax*, *Hypoponera* sp. Santschi, *Pheidole tolteca* Forel, *Dorymyrmex insanus* Buckley, *Pheidole* sp. 6, *Temnothorax augusti* Baroni Urbani and *Neivamyrmex sumichrasti* Norton) and 11 in both vegetation types (*Pheidole tepicana* Pergande, *Pheidole hirtula* Forel, *Linepithema dispertitum* Forel, *Pheidole nubicola* Wilson, *Temnothorax andrei*, *Monomorium ebenium* Forel, *Crematogaster* sp. Lund, *Temnothorax brevispinosus* Mackay W.P., *Camponotus* sp. 2, *Solenopsis pollux* Forel and *Labidus coecus* Latreille).

#### Dominance hierarchy MNP

For the dominance hierarchy experiment in the MNP, 648 baits were placed. Ants were recorded in 347 baits (53.5%), none were recorded in 263 (40.6%), and 38 baits (5.9%) were damaged by local fauna. Nine species of ants were recorded in the baits (*Formica* spp., *T. punctithorax*, *T. texanus* Wheeler, W.M., *Camponotus* spp., *M. mexicana*, *Monomorium minimum*, *Pheidole soritis*, *Pheidole* sp. 2, and *Lasius niger*). In the oak forest, six ant species were registered (*Formica* spp., *T. punctithorax*, *T. texanus*, *M. mexicana*, *M. minimum*, *P. soritis*), of which only three ant species were present in the ant-plant network. In the grassland, eight ant species were registered (*Formica* spp., *T. punctithorax*, *Camponotus* spp., *M. mexicana*, *M. minimum*, *P. soritis*, *Pheidole* sp. 2, and *L. niger*), of which only three ant species were present in the ant-plant network. Likewise, although *Crematogaster lineolata*, formed part of the network, it was not found at the baits.

The DI was determined only for the three ant species found in the baits and the oak forest network, i.e., *Formica* spp., *T. punctithorax,* and *M. mexicana*. The DI indicated that the behavior of all ant species was submissive (DI <0.5) (Table 1). Also, in the grassland, the DI was determined only for the three ant species found in the baits and the network, i.e., *Formica* spp., *T. punctithorax,* and *M. minimum*. The DI indicated that the behavior of all ant species was submissive (DI < 0.5). *M. minimum* was the least submissive of the grassland species, with a value of 0.31 (Table 1). In the oak forest, the DI of the core species in the network (*Formica* spp.) was not significantly different of the species at the periphery (*T. punctithorax* and *M. mexicana*) (DI Core: 0.04 ± 0.08, DI Periphery: 0.35 ± 0.50, *t* =  − 1.520, *P* = 0.1595). In the grassland, the DI of the core species in the network (*Formica* spp.) was significantly higher than of the species at the periphery (*T. punctithorax* and *M. minimum*) (DI Core: 0.06 ± 0.05, DI Periphery: 0 ± 0, *t* = 4.001, *df* = 13, *P* = 0.0015).

#### Dominance hierarchy FBSP

In the FBSP, ants were only registered in 170 out of the 504 baits (33.73%). Thirteen ant species were recorded on these baits (*M. ebenium*, *P. imparis*, *L. dispertitum*, *P. tepicana*, *C. rubrithorax*, *P. nubicola*, *D. insanus*, *T. andrei*, *T. rugithorax* Mackay, W.P., *T. augusti*, *T. brevispinosus*, *Nylanderia austroccidua* Trager and *Pheidole hirtula*). In the oak forest, of eight species registered at the baits (*M. ebenium*, *P. imparis*, *L. dispertitum*, *P. tepicana*, *T. andrei*, *T. rugithorax*, *T. brevispinosus,* and *P. hirtula*), only four species were registered in the ant-plant network (*P. imparis*, *L. dispertitum*, *P. hirtula,* and *M. ebenium*, Fig. 1A, Table 1). While in the grassland, of nine species registered at baits (*M. ebenium*, *L. dispertitum*, *C. rubrithorax*, *P. nubicola*, *P. tepicana*, *P. hirtula*, *D. insanus*, *T. augusti*, and *N. austroccidua*), only seven species were present in the ant-plant network (Fig. 1B, Table 1).

The DI was determined only for ants that were found both at the baits and in the network, i.e., in the oak forest, for *P. imparis, L. dispertitum, P. hirtula,* and *M. ebenium*; in the grassland, for *C. rubrithorax, M. ebenium, D. insanus, P. tepicana*, *P. nubicola*, *P. hirtula,* and *N. austroccidua* (Table 1). The DI indicated that all ant species behave as submissives (DI <0.5), with *M. ebenium* being the least submissive, with a value of 0.39 in the oak forest, and *D. insanus* with a value of 0.33 in the grassland (Table 1). In the oak forest, when comparing the DI of the core species of the network (*P. imparis*) with those of the periphery (*L. dispertitum, P. hirtula,* and *M. ebenium*), no significant differences were found (DI Core: 0.280 ± 0.208, DI Periphery: 0.245 ± 0.323, *t* = 0.259, *df* = 19, *P* = 0.7982). In the grassland, when comparing the DI of the core species of the network (*C. rubrithorax*) with those of the periphery (*M. ebenium, D. insanus, P. tepicana, P. nubicola, P. hirtula, and N. austroccidua*), no significant differences were found (*Z* =  − 0.573, *P* = 0.5914).

#### Modified dominance index MNP

In the oak forest of the MNP, the average MDI of the species was different from the DI, indicating that *Formica* spp. was the most dominant species (MDI = 0.78), and the behavior of the other species was submissive (MDI < 0.5) (Table 1). In the grassland, *Formica* spp. was also the most dominant species (MDI = 0.61), and the behavior of the other species was submissive (MDI <0.5). However, in the oak forest, no significant differences were found between the MDI of the core species of the network (*Formica* spp.) and those of the periphery (*T. punctithorax* and *M. minimum*) (MDI Core: 0.77 ± 0.081, MDI Periphery: 0.412 ± 0.472, *t* = 1.868, *df* = 10, *P* = 0.0913). Also, in the grassland, no significant differences were found between the MDI of the core species of the network (*Formica* spp.) and those of the periphery (*T. punctithorax* and *M. mexicana*) (MDI Core: 0.606 ± 0.138, MDI Periphery: 0.384 ± 0.374, *t* = 1.377, *df* = 13, *P* = 0.1918).

#### Modified dominance index FBSP

In FBSP, the average MDI of the species differed from the DI. In oak forest, *M. ebenium* was the species with the highest dominance value (MDI = 0.74), followed by *L. dispertitum* and *P. imparis* (which were also dominant with MDI values >0.5). Finally, *P. hirtula* had values <0.5, which classifies them as submissive species (Table 1). In the grassland, *P. nubicola* was the species with the highest dominance value (MDI = 1), followed by *C. rubrithorax*, *P. tepicana, D. insanus P. hirtula,* and *M. ebenium.* (which were also dominant with MDI values >0.5), and finally, *N. austroccidua* had values <0.5, which classifies them as a submissive species (Table 1). However, in the oak forest, no significant differences were found between the MDI of the core species (*P. imparis*) and those of the periphery (*L. dispertitum*, *P. hirtula,* and *M. ebenium*) of the network (MDI Core: 0.600 ± 0.36, MDI Periphery: 0.571 ± 0.42, *t* = 0.152, *df* = 19, *P* = 0.8809). Also, in the grassland, no significant differences were found between the MDI of the core species (*C. rubrithorax*) and those of the periphery (*M. ebenium*, *D. insanus*, *P. tepicana*, *P. nubicola*, *P. hirtula*, *and N. austroccidua*) of the network (MDI Core: 0.833 ± 0.16, MDI Periphery: 0.58 ± 0.39, *t* = 1.554, *df* = 22, *P* = 0.1345).

### Relationship of the dominance hierarchy and abundance in the structure of the ant-plant network

The nestedness ranking of the MNP’s oak forest includes the three species of ants present in both the network and baits. Thus, number one is the species with the highest number of links and number three, the species with the least number of links. The ranking of the MNP’s grassland includes three species of ants present in both the network and baits. In the FBSP’s oak forest, the ranking was from one to four, while in the grassland, the ranking was from one to seven. In neither vegetation type of the two study sites were significant associations found between the nestedness measures of the network (nestedness ranking, nestedness contribution, nested rank, and expected frequency) and the average DI of the ants (Table 2). Likewise, no correlation was found between the nestedness measures and abundance (measured by incidence at the baits) of the ants (Table 2). When we used the MDI, no significant relationship was found between the nestedness measures of the network and the average MDI of the ants (Table 2), except for the significant correlation found between the MDI and the Nc in the FBSP’s grassland (Table 2). When considering the pooled network for the MNP, no significant correlations were found between any of the nestedness measures, ant abundance or dominance hierarchy (Table S1).

## Discussion

We found similar patterns in the structure of the ant-plant interaction networks of the two temperate ecosystems (MNP and FBSP). The core ant species in the networks were both dominant and the most important (with the highest species strength value in their respective communities). However, the species’ position within the network was not correlated with their dominance hierarchy ranking or abundance, except in FBSP’s grassland, where a positive correlation was found between the ant dominance hierarchy and nested pattern (the MDI and Nc).

**Table 2:** Nestedness measures correlated with the abundance and dominance indices of the ants species registered at the Malinche National Park (MNP) and Flor del Bosque State Park (FBSP), for each vegetation type.

Nestedness measures	Rho	*Z*	*P*
**Ant species MNP Oak Forest**			
Nra-A	−0.500	−0.707	0.4795
Nra-DI	−0.500	−0.707	0.4795
Nra-MDI	−1.000	−1.414	0.1573
Nc-A	1.000	1.414	0.1573
Nc-DI	−0.500	−0.707	0.4795
Nc-MDI	0.500	0.707	0.4795
Nr-A	−1.000	−1.414	0.1573
Nr-DI	0.500	0.707	0.4795
Nr-MDI	−0.500	−0.707	0.4795
** Ant species MNP Grassland**			
Nra-A	−1.000	−1.414	0.1573
Nra-DI	0.500	0.707	0.4795
Nra-MDI	−0.500	−0.707	0.4795
Nc-A	−0.500	−0.707	0.4795
Nc-DI	1.000	1.414	0.1573
Nc-MDI	0.500	0.707	0.4795
Nr-A	−1.000	−1.414	0.1573
Nr-DI	0.500	0.707	0.4795
Nr-MDI	−0.500	−0.707	0.4795
**Ant species FBSP Oak Forest**			
Nra-A	−0.800	−1.386	0.1659
Nra-DI	0.200	0.346	0.7290
Nra -MDI	0.400	0.693	0.4884
Nc-A	0.200	0.346	0.7290
Nc-DI	0.800	1.386	0.1659
Nc-MDI	0.400	0.693	0.4884
Nr-A	−0.800	−1.386	0.1659
Nr-DI	0.200	0.346	0.7290
Nr-MDI	0.400	0.693	0.4884
Ef-A	−0.800	−1.386	0.1659
Ef-DI	0.200	0.346	0.7290
Ef-MDI	0.400	0.693	0.4884
** Ant species FBSP Grassland**			
Nra-A	−0.500	−1.225	0.2207
Nra-DI	−0.630	−1.543	0.1228
Nra -MDI	−0.700	0.4840	−0.286
Nc-A	0.036	0.087	0.9303
Nc-DI	0.148	0.363	0.7165
Nc-MDI	0.786	1.925	0.0543^*^
Nr-A	−0.643	−1.575	0.1153
Nr-DI	−0.482	−1.180	0.2379
Nr-MDI	−0.357	−0.875	0.3817
Ef-A	−0.482	−1.180	0.2379
Ef-DI	−0.500	−1.225	0.2207
Ef-MDI	−0.148	−0.363	0.7165

**Notes.**

Nestedness Ranking (Nra), Nestedness Contribution (Nc), Nested Rank (Nr), Expected frequency (Ef), Abundance (A), Dominance Index (*DI*), Modified Dominance Index (*MDI*).

* Significant *P* values.

Three of the four networks were nested; the only one that was not nested was the grassland network in MNP (Lara et al., 2020). In the MNP, in both types of vegetation, *Formica* spp. was identified as the most dominant (MDI) and important (with the highest species strength) core species of the ant-plant network. In FBSP, *Prenolepis imparis* and *Camponotus rubrithorax*, in the oak forest and grassland respectively, were identified as core, with the highest species strength and high dominance in these networks. In both types of vegetation, some ants in the network were not found in the baits (e.g., *Crematogaster lineolate* and *Pseudomyrmex pallidus*). Their nesting and feeding behaviors may explain their absence in the baits. Species of the genus *Pseudomyrmex* nest in rotten logs and the preformed cavities of living plants (Ward, 1990) and species belonging to the genus *Crematogaster* are characterized as omnivores that nest in trees and forage in plants and leaf litter (Moreira, Huising & Bignell, 2012). Behaviors such as these likely explain the absence of some species in the baits placed at ground level, which highlights the need to explore methods of recording ant species that forage on vegetation that reduce this bias.

Similar patterns are found in the four interaction networks. The asymmetry was comparable; there were more species of plants than of ants in all networks. Specialization asymmetry is also evident: the ants (especially in the grassland of the MNP) are more specialized than the plants. This asymmetry is expected when there are a higher number of plant species, as these provide different types of resources, in this case, honeydew, floral, and extrafloral nectar (Blüthgen & Fiedler, 2004). Specialization offers two advantages; it facilitates more efficient use of the available resources or reduces competition between the dominant ants of the community for those resources (Blüthgen et al., 2007), i.e., in MNP, *Formica* spp. or in FBSP, *P. imparis,* and *C. rubrithorax*. In networks such as these, the plants will be less specialized than the ants, possibly because ants offer a similar service (in the case of mutualism).

We consider that the modified hierarchy index proposed here better reflects the dominance hierarchy of the communities in both sites (MNP and FBSP) because otherwise, all the species would be considered submissive. In the MNP, the MDI reflects the dominance of *Formica* spp., above all, its spatial dominance due to its wide distribution (presence in 73% of the baits and 70% of the pitfall traps) (Table 1). This species’ dominance also coincides with that reported in other temperate environments, where different *Formica* species are recognized as the dominant species around which ant communities are structured (Czechowski et al., 2013; Trigos Peral et al., 2016). Another species with a high MDI was *Monomorium minimum*, which presented mass recruitment behavior. The ant species of this genus’ colonies are known to be moderate to large, containing up to 2000 workers and many queens (Wheeler, 1910), which could explain why *M. minimum* was best represented numerically in the baits. When the following species were found at the baits they were generally the only species present; *Formica* spp. (oak forest 77% of the baits, grassland 61%) and *M. minimum* (grassland 100%).This suggests that both these species tend to displace other ants from their food sources competitively.

In FBSP’s grassland, the MDI revealed *C. rubrithorax* to be the network’s dominant species and at its core. This species was also found occupying a higher number of baits without other species present (27 of 32 occupied baits) (Table 1). *C. rubrithorax* is characterized as a species that behaves aggressively for food resources, defends the plants it acquires its food from, and dominates in arid or semi-arid environments. It has ecological and physiological adaptations to withstand the high frequency of solar insolation and elevated daytime temperatures. This species travels short distances in search of food and selects resources based on their abundance (Guzmán-Mendoza & Castaño Meneses, 2007). In FBSP’s oak forest, *P. imparis* was found in the core of the network. This species’ MDI classifies it as dominant. Its tolerance of low temperatures and aggressive behavior explain its dominance in this zone (Cuautle, Vergara & Badano, 2016). In both vegetation types, some ant species present in the baits were not in the networks. The absence of these species may be because they are being displaced by more dominant species. For example, the ants of the genus *Temnothorax* are considered opportunistic foragers that flee when more aggressive species arrive (Prebus, 2017).

In three of the study sites (grassland and oak forest of MNP and, oak forest of FBSP), there was no correlation between the nestedness measures of the ants and their dominance hierarchy. So, in these communities, the dominance hierarchy does not predict the position of the ants within the network. However, in the FBSP’s grassland, a positive correlation was found between an ant’s MDI and its contribution to the nestedness pattern. Therefore, at least for one site, the ant’s position in the network seems to be determined by its dominance hierarchy rank. What distinguishes this site from the other three? Two factors might be determining this correlation: the ant richness and their behavioral dominance in this site. Research has cited network size as a possible determinant of network structure. Guimarães Jr et al. (2006) found that ant-extrafloral plant networks from tropical areas were nested, but those of temperate areas were not, and they mention that this could be attributed to the lower number of ant and plant species in the cloud forest they studied. The network in FBSP’s grassland had the highest number of ant species, whereas the number of ant species registered at the other three sites was much lower, especially MNP’s networks. Most ant-plant network studies have been carried out in the neotropics (80%); besides their nested structure, a characteristic shared by these tropical networks is that they are species-rich (Del-Claro et al., 2018). The other factor that stands out in the grassland of the FBSP is the dominance hierarchy values of its ant community. Although not significantly different, the mean MDI of this ant community was higher than the mean MDI of the other ant communities (mean ± sd, MNP: Oak forest = 0.31 ± 0.41, Grassland = 0.41 ± 0.21; FBSP: Oak forest = 0.56 ± 0.21, Grassland = 0.60 ± 0.32). The ant community in FBSP’s grassland contains a considerable number of the subordinate camponotini (*C. rubrithorax*) and the generalist myrmicinae functional group (*Pheidole* spp. and *M. ebenium*). As mentioned above, *C. rubrithorax* is characterized by its dominant behavior, especially in open areas such as grasslands (Andersen 200). Generalist mymicinae are characterized by their abundance, mass recruitment, and high competitive ability, mainly in open habitats (Andersen, 2000). These characteristics, plus the species richness and higher dominance values, make FBSP’s ant community more similar to those found in tropical habitats where the correlation between the DI and nestedness has been positive (Dáttilo, Díaz-Castelazo & Rico-Gray, 2014; Dáttilo et al., 2014a). Of the four nestedness measures used, a positive correlation was observed just with the Nc, likely because this measure better represents heterogeneity in the degree distribution of the ants. *C. rubrithorax* and *Pseudomyrmex* sp. had a higher degree distribution than the rest of the ants; therefore, their contribution to network nestedness is higher. In the other three of our study sites, the dominance hierarchy has little or no influence on the structure of the ant-plant network. In the MNP, both networks were species-poor networks.

We would like to point out a tacit, although untested, assumption of our research, that the interactions between the ants and the plants are mutualistic. However, the nested pattern present in three of the four networks lends weight to the hypothesis that they are mutualistic interactions, as nestedness is the characteristic pattern of mutualistic networks (Bascompte et al., 2003; Bascompte & Jordano, 2007). Nevertheless, the lack of correlation between the ants’ position in the network and its dominance could be explained by other interaction types, such as commensalistic interactions or even antagonism. Specific experiments (removal of ants from the plants) need to be carried out to test the interaction outcomes, at least of those with the highest interaction strength.

Although *Formica* spp. was the most dominant species and was found in the core of the oak forest and the grassland networks, the submissive species present in the periphery were not ordered in the network (nested ranking) according to their DI or MDI; this is true, even for the network aggregating both habitats (see Methodology and Table S1). In our study sites, the difference in dominance indices is significant, with no gradient (i.e., hierarchy) observed between *Formica* spp. and the submissive species or within the submissive species. In FBSP’s oak forest, the dominant ant *P. imparis* was found in the core of the network. However, the submissive species present in the periphery did not show the ordering in the network that was expected given their MDI values. Several factors could explain the lack of concordance between the nestedness measures and ant dominance hierarchy. In temperate ecosystems, there is less richness of ant species, which has been shown to reduce the importance of competition between species (Arnan et al., 2018). When ant communities are dominated by just a few species (mainly cold climate specialists such as *P. imparis* and *Linepithema dispertitum*), these species may diminish gradient in the dominance hierarchy. Other factors such as humidity and temperature could also come into play (García-Pérez et al., 1992; Flores-Maldonado, Phillips & Sánchez-Ramos, 1999; Rojas & Fragoso, 2000). For example, soil conditions seem to determine the network structure of mutualistic ant-plant networks, as increased nestedness is observed in soils with pH close to neutral (Dáttilo, Guimarães Jr & Izzo, 2013). The strong weathering and torrential rains leach nutrients from tropical soils, making them more impoverished than temperate forests (Tagami, Twining & Wasserman, 2012). The higher temperatures and precipitation in tropical environments affect plant growth negatively. Meanwhile, biological organisms tend to grow faster in these conditions generating increased competition for the available resources (Tagami, Twining & Wasserman, 2012). The scarcity of resources in tropical ecosystems could result in higher reciprocal selective pressure between ants and plants than in temperate environments. Therefore, the more relaxed competition in our temperate study zone may be a factor that contributes to the lack of correlation between ant network position and dominance hierarchy.

For both temperate sites, the number of ant species interacting in the network is small compared to the plant species. This disparity in the number of plants than in ant species could indicate there are sufficient food resources (in this case, plants) to cover the requirements of the interacting species that feed on floral nectar, extrafloral nectar, or honeydew, allowing the ants to specialize in one of these resources. Alternatively, species that are omnivorous (that do not depend exclusively on nectar) or granivorous (i.e., *M. minimum*) could be reducing competitive interaction. This may explain why *M. minimum,* which occupies the second position in the dominance hierarchy (MDI) and is associated with only one species of plant in the MNP’s grassland, does not have to compete with *Formica* spp., which must forage in many species of plants to meet its needs for nectar and honeydew (Trigos Peral et al., 2016). However, the possibility that *Formica* spp. is displacing other species of ants that use nectar as their primary resource (i.e., genera *Camponotus* spp. and *C. lineolata*) cannot be entirely ruled out.

In conclusion, the dominance hierarchy recorded in our study is not as pronounced as in tropical zones (Dáttilo, Díaz-Castelazo & Rico-Gray, 2014), where there is a greater richness of ant species (Mackay et al., 1991) and more competition (Dáttilo, Díaz-Castelazo & Rico-Gray, 2014; Arnan et al., 2018). No correlation was found between the ant abundance and nestedness measures. Although the core ant species had the highest number of incidences in pitfall traps (Table 1), the incidence values of the other species did not produce the expected ordering in nestedness measures. This discrepancy indicates that abundance has little or no influence on network structure in temperate ecosystems, unlike tropical areas (Dáttilo, Díaz-Castelazo & Rico-Gray, 2014). Correspondingly, other studies have reported that species’ abundance does not explain the frequency of interactions or network structure. For example, Díaz-Castelazo et al. (2004) evaluated whether the abundance of ants and plants with EFNs affected the frequency of ant-plant interactions (using baits and measuring the frequency of interactions on plants). They found that the proportion of associated ant and plant species was consistently higher than that of non-associated ant and plant species (those species that were non-interacting but present at baits or having EFNs). This finding indicates that EFN mediated ant-plant associations are not merely the effect of an abundance or richness of plants with EFNs and nectarivorous ants. Other studies that analyze ecological networks have found that the interactions and network structure cannot be explained solely by the abundance of species (Vázquez et al., 2009; Ramos-Robles, Andresen & Díaz-Castelazo, 2016). The dominant species in FBSP, *P. imparis* (Sorrells et al., 2011) and *C. rubrithorax* (Rico-Gray et al., 1998), as well as *Formica* spp. in MNP (Spotti et al., 2015), are ants that usually feed on liquids derived from plants or honeydew. They are likely monopolizing these plant resources, which decreases the importance of the other ant species in the network.

## Conclusions

Our results suggest that in temperate ecosystems, ant abundance and dominance hierarchy have little influence on the structure of ant-plant networks. Therefore, the main drivers of network structure in these ecosystems are likely to be other biotic factors (e.g., distributional patterns, Díaz-Castelazo et al., 2004; ant body mass, Kaspari & Weiser, 1999; ant food preferences and the presence of other species that interrupt mutualistic interactions, Díaz-Castelazo et al., 2010; Dáttilo et al., 2014a) or abiotic factors (e.g., temperature, precipitation, Rico-Gray et al., 2012; soil pH, Dáttilo, Guimarães Jr & Izzo, 2013). However, in the ant-plant network for which the correlation was positive, the richer ant-plant network and a more dominant ant community might very well be the factors underlying this result. Further research of interaction networks formed in temperate environments is needed to fully understand the architecture of the biodiversity they harbor. To our knowledge, this is the first study that evaluates the contribution of dominance hierarchy to the nested topology of ant-plant networks in temperate environments. This study highlights that the ant-plant networks of temperate environments do not respond to the same biotic factors than those of tropical environments, and opens a new line of research into these networks in particular and plant-animal interactions in general.

##  Supplemental Information

10.7717/peerj.10435/supp-1 Table S1 Nestedness measures correlated with the abundance and dominance indices of the ants species registered at the Malinche National Park (MNP) (the data from both types of vegetation was pooled) Nestedness Ranking (Nra), Nestedness Contribution (Nc), Nested Rank (Nr), Expected frequency (Ef), abundance (A), Dominance Index (*DI*), Modified Dominance Index (*MDI*)

10.7717/peerj.10435/supp-2 Table S2 Ant and plant species of the ant-plant interaction networks in Flor del Bosque State Park (FBST) Oak forest (OF, Fig. 1A) and grassland (G, Fig. 1B).

10.7717/peerj.10435/supp-3 Table S3 Raw Data Frequency Network FBSP Raw data of the frequency of the ant-plant interaction matrix in FBST (oak forest and grassland)

## Additional Information and Declarations

### Field Study Permissions

The following information was supplied relating to field study approvals (i.e., approving body and any reference numbers): The Secretaria de Medio Ambiente y Recursos Naturales (SEMARNAT) (Secretary of Environment and Natural Resources) approved the collection permit SGPA/DGV/06901/15 for this study.

### Data Availability

The following information was supplied regarding data availability: The raw data is available as Supplemental File.
