# Supplementary material for: Neither ant dominance nor abundance explain ant-plant network structure in Mexican temperate forests
Source: PeerJ. 2020 Dec 7;8:e10435. doi: 10.7717/peerj.10435 (PMC7727367; doi:10.7717/peerj.10435)
Supplement: Table S1 — Nestedness Ranking (Nra), Nestedness Contribution (Nc), Nested Rank (Nr), Expected frequency (Ef), abundance (A), Dominance Index (DI), Modified Dominance Index (MDI) [file peerj-08-10435-s001.docx]

| Nestedness measures | Rho | Z | *P* |
| --- | --- | --- | --- |
| Ant species MNP |  |  |  |
| Nra-A | -0.700 | -1.400 | 0.1615 |
| Nra-DI | -0.075 | 0.150 | 0.8808 |
| Nra-MDI | -0.200 | -0.400 | 0.6892 |
| Nc-Sa | -0.100 | -0.200 | 0.8415 |
| Nc-DI | 0.825 | 1.650 | 0.0989 |
| Nc-MDI | 0.100 | 0.200 | 0.8415 |
| Nr-Sa | -0.700 | -1.400 | 0.1615 |
| Nr-DI | 0.075 | 0.150 | 0.8808 |
| Nr-MDI | -0.200 | -0.400 | 0.6892 |
| Ef-Sa | -0.667 | -1.334 | 0.1823 |
| Ef -DI | -0.132 | -0.263 | 0.7924 |
| Ef -MDI | -0.154 | -0.308 | 0.7582 |
