# Supplementary material for: Neither ant dominance nor abundance explain ant-plant network structure in Mexican temperate forests
Source: PeerJ. 2020 Dec 7;8:e10435. doi: 10.7717/peerj.10435 (PMC7727367; doi:10.7717/peerj.10435)
Supplement: Table S2 — Oak forest (OF, Fig. 1A) and grassland (G, Fig. 1B). [file peerj-08-10435-s002.docx]

| Plants FBSP OF |  |  | Plants FBSP G |  |  | |
| --- | --- | --- | --- | --- | --- | --- |
| Family/Subfamily | Genus/Species | Codes | Family/Subfamily | Genus/Species | Codes | |
| Agavaceae | *Agave salmiana* | AGSA | Agavaceae | *Agave potatorum* | | AGPO |
| Agavaceae | *Agave* | AGA6 | Agavaceae | *Agave* | | AGA1 |
| Amaryllidaceae | *Sprekelia formosissima* | SPFO | Agavaceae | *Agave* | | AGA2 |
| Anacardiaceae | *Rhus standleyi* | RHST | Agavaceae | *Agave* | | AGA3 |
| Apocynaceae | *Metastelma angustifolium* | MEAN | Agavaceae | *Agave* | | AGA4 |
| Asteraceae |  | AST1 | Agavaceae | *Agave* | | AGA5 |
| Asteraceae | *Perymenium mendezii* | PEME | Asclepiadaceae |  | | ASCL |
| Asteraceae | *Eupatorium deltoideum* | EUDE | Asteraceae | *Baccharis salicifolia* | | BASA |
| Asteraceae | *Gnaphalium* | GNAP | Asteraceae | *Perymenium mendezii* | | PEME |
| Asteraceae | *Senecio multidentatus* | SEMU | Asteraceae | *Eupatorium deltoideum* | | EUDE |
| Asteraceae | *Barkleyanthus salicifolius* | BASL | Asteraceae | *Barkleyanthus salicifolius* | | BASL |
| Asteraceae | *Verbesina virgata* | VEVI | Asteraceae | *Senecio* | | SENE |
| Bromeliaceae | *Bromelia* | BROM | Asteraceae | *Stevia serrata* | | STSE |
| Cactaceae | *Opuntia* | OPU1 | Cactaceae | *Opuntia huajuapensis* | | OPHU |
| Commelinaceae | *Commelina* | COM1 | Cactaceae | *Opuntia* | | OPU1 |
| Convolvulaceae | *Ipomoea* | IPO1 | Cactaceae | *Opuntia* | | OPU2 |
| Convolvulaceae | *Ipomoea* | IPO2 | Commelinaceae | *Commelina* | | COM2 |
| Convolvulaceae | *Ipomoea stans* | IPST | Fabaceae | *Eysenhardtia polystachya* | | EYPO |
| Convolvulaceae | *Ipomoea hematica* | IPHE | Fabaceae | *Eysenhardtia* | | EYSE |
| Cyperaceae | *Cyperus* | CYPE | Fabaceae | *Mimosa aculeaticarpa* | | MIAC |
| Fabaceae | *Brongniartia intermedia* | BRIN | Loranthaceae |  | | LORA |
| Fabaceae | *Calliandra grandiflora* | CAGR | Myrtaceae | *Eucalyptus* | | EUCA |
| Fabaceae | *Cologania obovata* | COOB | Passifloraceae | *Passiflora exsudans* | | PAEX |
| Fabaceae | *Mimosa aculeaticarpa* | MIAC | Poaceae |  | | POAC |
| Fagaceae | *Quercus mexicana* | QUME | Polygalaceae | *Monnina schlechtendaliana* | | MOSC |
| Lamiaceae | *Salvia polystachya* | SAPO | Rosaceae | *Amelanchier denticulata* | | AMDE |
| Lilaceae |  | LILA | Sapindaceae | *Dodonaea viscosa* | | DOVI |
| Orobanchaceae | *Castilleja* | CAST | Solanaceae | *Solanum nigrescens* | | SONI |
| Passifloraceae | *Passiflora exsudans* | PAEX |  |  | |  |
| Poaceae | *Tripsacum dactyloides* | TRDA |  |  | |  |
| Polygalaceae | *Monnina ciliolata* | MOCI |  |  | |  |
| Rhamnaceae | *Ceanothus caeruleus* | CECA |  |  | |  |
| Rosaceae | *Amelanchier denticulata* | AMDE |  |  | |  |
| Asteraceae |  | AST2 |  |  | |  |
| Ants FBSP OF |  |  | Ants PEFB G |  | |  |
| Dolichoderinae | *Linepithema dispertitum* | LIDI | Dolichoderinae | *Dorymyrmex insanus* | | DOIN |
| Formicinae | *Prenolepis imparis* | PRIM | Formicinae | *Camponotus rubrithorax* | | CARU |
| Myrmicinae | *Crematogaster* | CREM | Formicinae | *Nylanderia austroccidua* | | NYAU |
| Myrmicinae | *Monomorium ebenium* | MOEB | Formicinae | *Prenolepis imparis* | | PRIM |
| Myrmicinae | *Pheidole hirtula* | PHHI | Myrmicinae | *Monomorium ebenium* | | MOEB |
| Myrmicinae | *Pheidole nubicola* | PHNU | Myrmicinae | *Pheidole tepicana* | | PHTE |
| Myrmicinae | *Temnothorax tricarinatus* | TETR | Myrmicinae | *Pheidole hirtula* | | PHHI |
| Pseudomyrmecinae | *Pseudomyrmex pallidus* | PSPA | Myrmicinae | *Pheidole nubicola* | | PHNU |
| Pseudomyrmecinae | *Pseudomyrmex* | PSEU | Myrmicinae | *Pheidole* | | PHEI |
|  |  |  | Myrmicinae | *Temnothorax* | | TEMN |
|  |  |  | Pseudomyrmecinae | *Pseudomyrmex pallidus* | | PSPA |
|  |  |  | Pseudomyrmecinae | *Pseudomyrmex* | | PSEU |
